# Hepatitis B viral core protein disrupts human host gene expression by binding to promoter regions

**DOI:** 10.1186/1471-2164-13-563

**Published:** 2012-10-22

**Authors:** Yanhai Guo, Wei Kang, Xiaoying Lei, Yongnian Li, An Xiang, Yonglan Liu, Jinrong Zhao, Ju Zhang, Zhen Yan

**Affiliations:** 1 State Key Laboratory of Cancer Biology, Department of Pharmacogenomics, School of Pharmacy, Fourth Military Medical University, 169 West Changle Road, Xi’an, 710032, China; 2 Department of Clinical Laboratory, Affiliated Hospital of Xi’an Medical University, Xi’an, 710077, China; 3 Department of Infectious Diseases, 323 Hospital, Xi’an, 710000, China

**Keywords:** Hepatitis B virus, Hepatitis B core protein, Chromatin immunoprecipitation microarray, ChIP-on-chip, Gene expression, DNA-protein interaction

## Abstract

**Background:**

The core protein (HBc) of hepatitis B virus (HBV) has been implicated in the malignant transformation of chronically-infected hepatocytes and displays pleiotropic functions, including RNA- and DNA-binding activities. However, the mechanism by which HBc interacts with the human genome to exert effects on hepatocyte function remains unknown. This study investigated the distribution of HBc binding to promoters in the human genome and evaluated its effects on the related genes’ expression.

**Results:**

Whole-genome chromatin immunoprecipitation microarray (ChIP-on-chip) analysis was used to identify HBc-bound human gene promoters. Gene Ontology and pathway analyses were performed on related genes. The quantitative polymerase chain reaction assay was used to verify ChIP-on-chip results. Five novel genes were selected for luciferase reporter assay evaluation to assess the influence of HBc promoter binding. The HBc antibody immunoprecipitated approximately 3100 human gene promoters. Among these, 1993 are associated with known biological processes, and 2208 regulate genes with defined molecular functions. In total, 1286 of the related genes mediate primary metabolic processes, and 1398 encode proteins with binding activity. Sixty-four of the promoters regulate genes related to the mitogen-activated protein kinase (MAPK) pathways, and 41 regulate Wnt/beta-catenin pathway genes. The reporter gene assay indicated that HBc binding up-regulates proto-oncogene tyrosine-protein kinase (SRC), type 1 insulin-like growth factor receptor (IGF1R), and neurotrophic tyrosine kinase receptor 2 (NTRK2), and down-regulates v-Ha-ras Harvey rat sarcoma viral oncogene (HRAS).

**Conclusion:**

HBc has the ability to bind a large number of human gene promoters, and can disrupt normal host gene expression. Manipulation of the transcriptional profile in HBV-infected hepatocytes may represent a key pathogenic mechanism of HBV infection.

## Background

Hepatitis B virus (HBV) infection is a serious public health threat. Worldwide, more than 400 million people are infected with HBV. Chronic infection is associated with a high risk of potentially life-threatening liver diseases, including hepatitis, cirrhosis, and hepatocellular carcinoma [1,2]. However, despite extensive research efforts, the molecular mechanisms of chronic HBV infection remain to be fully elucidated. HBV, a hepatotropic virus, is a 3.2 kb partially double-stranded DNA virus that replicates through the reverse transcription of pre-genomic RNA. Its DNA genome contains four overlapping open reading frames (ORFs), which encode the surface antigen-associated S proteins (large S, middle S, and major S), core antigen proteins (precore and core (HBc)), reverse transcriptase (P), and the multifunctional X protein (HBx) [3]. Diverse molecules and proteins may participate in HBV-infection induced chronic hepatitis. For example, a recent study indicated that the cytokines lymphotoxin (LT) alpha and beta and their receptor (LTbetaR) are upregulated in HBV-induced hepatitis and hepatocellular carcinoma (HCC) [4]. While the HBV proteins play essential roles in viral processes, such as virulence and replication, they also exert direct effects on host cellular functions to promote virus survival. For instance, HBV targets host genes that are involved in cell survival, and while this facilitates escape from immune surveillance and clearance, it also favors malignant transformation [5].

One of the possible mechanisms that contributes to malignant transformation involves HBV-encoded trans-activating factors, which influence particular intracellular signal transduction pathways by altering the host gene expression profile in hepatocytes [6]. Several HBV protein products have been characterized as modulators of cellular growth, repair, and death, all of which are involved in oncogenesis [7]. Studies have shown that HBx can transactivate the expression of all HBV proteins, including HBc, which acts as the core antigen that stimulates the human immune response. The 21–22 kDa HBc protein has been detected in both the nuclear and cytoplasmic compartments of hepatocytes infected by HBV [8,9]. Subsequent studies have revealed pleiotropic functions of HBc that affect host processes, including the malignant transformation of chronically infected liver cells [10]. Moreover, this newly-recognized dual character of HBc as a novel regulator of the HBV life cycle and of hepatocellular carcinogenesis has been hypothesized to involve HBx, possibly through an inhibitory feedback mechanism [11]. HBc was shown to repress the expression of the human tumorigenesis-associated genes, interferon (IFN)-β and p53 [12]; the latter of which is also modulated by HBx, *via* binding to the encoded protein and suppressing its activity [13]. Additionally, recent studies have suggested that HBV down-regulates the human IFN-inducible MxA promoter through direct interaction of precore/core proteins [14], and have shown that HBc inhibits apoptosis induced by the tumor necrosis factor family member, TRAIL, in hepatocytes by blocking gene expression of the pathway-related death receptor [10]. Collectively, these studies indicate that HBc may interact with the human genome to modulate normal functions of liver cells infected HBV.

HBc is the major capsid protein of the virus and self-assembles to form the subviral 30–32 nm nucleocapsid particles, which package the viral polymerase and pre-genomic RNA during RNA replication. The HBc carboxy-terminus contains two nuclear localization signals in regions of arginine-rich sequences, and an SPRRR motif with several serine residues that are targets of phosphorylation [15]. In general, the C-terminus amino acid sequence is rich in basic amino acid residues, such as arginine and lysine, and resembles the structure of a mammalian DNA binding protein, protamine [16,17]. Although HBc has been sufficiently demonstrated by many studies to functionally bind to both virus- and host-derived RNA and DNA, the way in which HBc interacts with the human genome to modulate normal hepatocyte function in HBV infection remains unknown. Based on the findings of *in vivo* studies that showed core particles binding specifically to the HBV pregenome and genome, we hypothesized that HBc may also bind specifically to certain human gene promoters, either through its C-terminal functional domain or its N-terminal assembling domain.

A recently developed high-throughput strategy to perform targeted or genome-wide studies of transcription binding factors is the chromatin immunoprecipitation (ChIP)-coupled DNA microarray analysis. Known as ChIP-on-chip, this technique couples immunoprecipitation of chromatin-bound transcription factors with the identification of bound DNA sequences through hybridization on DNA microarrays [18]. In the present work, we used a combination of ChIP and location analysis with genome-wide tiling arrays to generate a human genome-wide binding profile of HBc. The human genes whose promoters were bound by HBc and had functions related to tumorigenesis were selected for verification by quantitative PCR (qPCR) and functional analysis by gene expression assays. Identification of the human gene targets of the HBV-encoded HBc protein provides further insights into HBV pathogenesis and potential new targets of molecular therapeutics against HBV-associated hepatocellular carcinogenesis.

## Methods

### Research subjects and biological samples

Thirteen unrelated patients, including nine males and four females ranging in age from 19 to 55 years-old (mean: 33.5), diagnosed with chronic hepatitis B (CHB) were recruited to our study. Three male healthy blood donors, whose age and gender matched the patient group, were enrolled as controls. The diagnostic criteria of CHB was based on the combination of clinical history, physical examination, imaging and laboratory data, and/or histology, according to the published guidelines of the Chinese Medical Association [19]. All patients were also evaluated to ensure that the following criteria were satisfied: (1) serum hepatitis B surface antigen (HBsAg), hepatitis B e antigen (HBeAg), and hepatitis B core antibody (HBcAb) (IgG) positive, but hepatitis B surface antibody (HBsAb) and hepatitis B e antibody (HBeAb) negative; (2) HBsAg and HBcAb positivity for over 12 months; (3) HBV DNA ≥3log10 copies/mL; (4) at least a six-month history of no type of antiviral, immuno-suppressive, or immunomodulatory treatment; (5) no evidence of co-infection with human immunodeficiency virus, hepatitis A virus, hepatitis C virus, or hepatitis D virus; (6) no routine alcohol consumption, by self-report.

A percutaneous liver biopsy was obtained from all study participants by using a 16 G Tru cut soft tissue biopsy needle. A ~176 mg specimen of normal liver tissue was obtained from the three healthy controls. A 9.3 ~ 19.0 mg total weight specimen of benign liver tissue was obtained from each CHB patient through a sequence of two to four biopsies. All liver samples were evaluated by histological examination, and stored in liquid nitrogen until further use. The clinical characteristics of patients and their corresponding biological samples are listed in Table 1. All subjects provided written informed consent to participate in the study, and the experimental protocol was approved by the Ethics Committees of the 323 Hospital and Affiliated Hospital of Xi’an Medical College (both of Xi’an, China).

**Table 1:** Clinical characteristics of study participants and their corresponding biological samples

**Number**		**Sex**	**Age**	Disease**	**HBV DNA (copies/mL)**	**Tissue weight (mg)**	
Pool*	**Samples**					**Sample**	**Pool**
1	1	male	30	CHB	4.5 × 10^5^	16.8	42.1
	2	male	27	CHB	5.7 × 10^6^	16.0	
	3	male	31	CHB	4.2 × 10^6^	9.3	
2	4	male	21	CHB	3 × 10^5^	17.0	43.6
	5	male	34	CHB	7.5 × 10^5^	12.1	
	6	male	25	CHB	3.2 × 10^6^	14.5	
3	7	male	40	CHB	3 × 10^4^	14.3	42.1
	8	male	39	CHB	8.3 × 10^5^	14.8	
	9	male	35	CHB	1.34 × 10^6^	13.0	
4	10	female	43	CHB	3.6 × 10^5^	10.9	48.6
	11	female	45	CHB	3.7 × 10^7^	17.8	
	12	female	40	CHB	6.9 × 10^7^	9.7	
	13	female	41	CHB	4.2 × 10^6^	10.2	
5	14	male	31	control	undetected	81.0	176.0
	15	male	30	control	undetected	50.0	
	16	male	25	control	undetected	45.0	

* Liver biopsy samples from three to four individuals were pooled into a single specimen for ChIP-on-chip and ChIP-qPCR analyses.

** CHB, chronic hepatitis B. The serum HBV markers used for diagnosis were: HBsAg(+), HBsAb(−), HBeAg(+), HBeAb(−), and anti-HBc(+). Healthy controls were confirmed as: HBsAg(−), HBsAb(−), HBeAg(−), HBeAb(−), and anti-HBc(−).

### Chromatin immunoprecipitation microarrays and data analysis

The hepatocytes isolated from pooled tissue samples (CHB, n = 4; control, n = 3) were cross-linked with formaldehyde and subjected to the standard ChIP procedure, as described elsewhere [20]. Briefly, the cross-linked hepatocytes were lysed, and nuclear extracts were sonicated to solubilize the protein-DNA complexes. The supernatants were then incubated overnight with mouse antibodies against the HBV core protein or normal mouse IgG (negative control) (both from Santa Cruz Biotechnology, Santa Cruz, CA, USA). The immuno-complexes were precipitated by addition of protein G-Sepharose (GE Healthcare, formerly Amersham Biosciences, Picastaway, NJ, USA), and eluted by incubation with the corresponding elution buffer. The cross-links were reversed by heating at 65°C for 5 h. The released proteins were digested with proteinase K, and the precipitated chromatin was extracted using a standard phenol-chloroform method.

The precipitated chromatin samples were sent to Kangcheng Biological Engineering Ltd., Comp. (Shanghai, China) for hybridization to the NimbleGen HG18 RefSeq Promoter Microarray (Roche Nimblegen, Madison, WI, USA). The immunoaffinity-enriched DNA was amplified for labeling using the GenomePlex® Whole Genome Amplification kit (Sigma-Aldrich, St. Louis, MO, USA), according to the manufacturer’s protocol. The amplified DNA samples were purified with a QIAquick spin kit (Qiagen, Valencia, CA, USA). The purified DNA was quantified using a Nanodrop ND-1000 spectrophotometer (Nanodrop Technologies, Wilmington, DE, USA). For DNA labeling, as previously described [21], the NimbleGen Dual-Color DNA labeling kit was used, according to the manufacturer’s guidelines detailed in the NimbleGen ChIP-on-chip protocol. A permutation-based peak-finding algorithm, NimbleScan (v2.5; NimbleGen), was used to identify peaks that represent significant positive enrichment. NimbleScan detects peaks by searching for four or more probes producing signals that were above the specified cutoff values (ranging from 90% to 15%) in a given 500 bp sliding window. The cutoff values were defined as a percentage of a hypothetical maximum, which was the mean + 6 [standard deviation]. The ratio data was then randomized 20 times to evaluate the probability of “false positives”. Each peak was then assigned a false discovery rate (FDR) score based on the randomization. The lower the FDR score, the more likely the peak was considered to correspond to a protein binding site.

To monitor the possible interference by non-specific hybridization signals of HBV DNA and promoter microarray probes, the amplified PCR products of HBV DNA genomic sequences were hybridized to the promoter microarray probes. HBV DNA was extracted from the serum (HBV DNA 6.0 × 10^8^ copies/mL) of HBV-infected patients using the standard phenol-chloroform method. Six overlapping regions of HBV genomic sequence were amplified by PCR, and the length of these products ranged from 534 bp to 654 bp. The sequences represent the entire HBV genome, with each two neighboring fragments overlapping by 79–99 bp of sequence (see Additional file 1). The PCR products were sent to Kangcheng Co. for hybridization to the NimbleGen HG18 RefSeq Promoter Microarray.

### Quantitative polymerase chain reaction (qPCR)

The results from the ChIP-on-chip microarray analysis were verified by qPCR. The reaction was carried out using an iCycler iQ real-time PCR detection system and the iQ SYBR Green Supermix detection reagent (Bio-Rad, Hercules, CA, USA). Gene-specific primers were designed with the Beacon Designer software (Premier Biosoft International, Bio-Rad) and are listed in Additional file 2. Relative expression was normalized as a percentage of input (total chromosomal DNA). To account for chromatin sample preparation differences, each output DNA fraction Ct value was normalized to the input DNA fraction Ct value for the same qPCR assay (ΔCt). Relative quantification was performed by the Livak (2^-△△Ct^) method [22]. The % input for each output fraction was calculated according to the formula: %Input = [2(CtInput-CtOutput) × Fd] × 100%, where Fd is the input dilution factor).

### Luciferase reporter assay

Genomic fragments (250 ~ 500 bp) encompassing a single HBc peak from the promoter region of selected genes were amplified by PCR from human genomic DNA and cloned into pGL3-basic expression vectors (Promega, Madison, WI, USA) (see Additional file 3). The HBc expression plasmid (pUSC) was constructed as previously described [23], for use in co-transfection assays. The inserted sequences of all reporter constructs were verified by direct sequencing.

HepG2 cells were transfected with 1000 ng of one of the pGL3-basic constructs plus 800 ng of the pUSC plasmid, together with 20 ng pRL-TK vector (Promega) as an internal control for transfection efficiency, using the JetPEI transfection reagent (PolyPlus-Transfection Co., Illkirch, France). After 24 hours, cells were collected and luciferase activities were measured using the Dual-Luciferase Reporter Assay system (Promega). Firefly luciferase activities were normalized to the level of Renilla luciferase activity in each transfection experiment. Relative luciferase levels were expressed as the average of three independent experiments performed in quadruplicate.

### Data analysis

Raw microarray data were extracted as pair files by the NimbleScan software. We performed median-centering, quantile normalization, and linear smoothing by using the Bioconductor packages, Ringo, Limma, and MEDME. The gene ontology (GO) and pathway analysis of promoter-related genes were carried out online ( http://www.geneontology.org). Gene pathway analysis was conducted with the Kyoto Encyclopedia of Genes and Genomes (KEGG) collection of online databases dealing with genomes, enzymatic pathways, and biological chemicals [24].

Statistical analysis was performed using SPSS software (v9.0; SPSS Inc., Chicago, IL, USA). Statistically significant differences among continuous variables between two independent groups were analyzed by the Mann–Whitney test. A *p*-value less than 0.05 was considered statistically significant.

## Results

### Genome-wide binding profile of HBc in HBV-infected hepatocytes

We performed ChIP-on-chip analysis with the NimbleGen HG18 RefSeq Promoter Microarray consisting of 18028 of the best defined human transcript-associated promoters. Hepatocytes infected with HBV and expressing HBV core protein were isolated from CHB patients, immunoprecipitated with HBc antibody, and investigated to determine the genome-wide profile of bound host gene promoters. IgG pre-incubated with 100-fold excess HBc antibody was used as a negative control, and immunoprecipitation of hepatocyte cells from healthy individuals was used as a blank control. Meanwhile, no non-specific hybridization signals between the HBV DNA and the chip probes (NimbleGen HG18 RefSeq Promoter Microarray) were detected. The HBV-infected hepatocyte-specific enrichment that was achieved by sequential immunoprecipitations was expected to facilitate a sufficiently comprehensive identification of HBc binding sites, including the relatively weakly bound sites.

In total, 3100 HBc-immunoprecipitated host promoter regions were found to be enriched by at least 2-fold in HBc-infected tissues compared to the blank controls (*p* < 0.05) (see Additional file 4). Among these, 2607 (84.10%) were located in high CpG density promoters (HCPs), 356 (11.50%) in intermediate CpG density promoters (ICPs), and 137 (4.42%) in low CpG density promoters (LCPs). Ninety-two percent (2833) of the differentially enriched gene promoter regions were immunoprecipitated from all four of the pooled samples evaluated. These results suggest that the HBV core protein might bind to a broad spectrum of human gene promoters, especially those featuring high CpG density.

**Figure 1:**
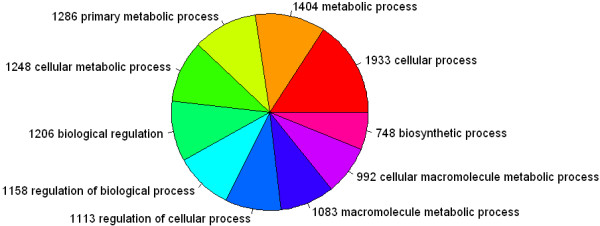
**Count proportion pie of the GO analysis of Biological Process**. The chart shows the top ten counts of the significant enrichment terms of designated gene involving Biological Process

**Figure 2:**
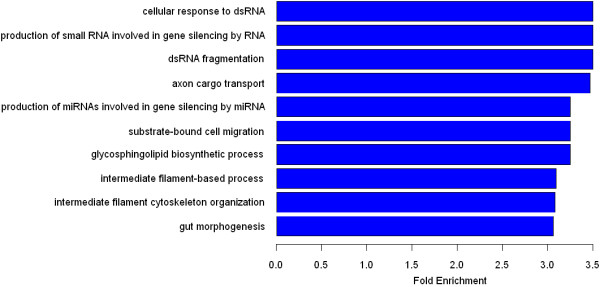
**The bar plot of the GO analysis of Biological Process.** The bar plot shows the top ten Fold Enrichment value of the significant enrichment terms of designated gene involving Biological Process

**Figure 3:**
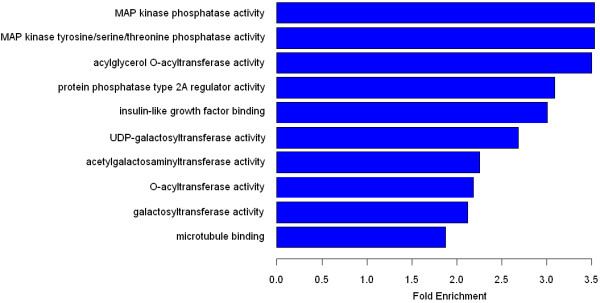
**The bar plot of the GO analysis of Molecular Function.** The bar plot shows the top ten Fold Enrichment value of the significant enrichment terms of designated gene involving Molecular Function

The GO analysis is a powerful tool by which the 3100 genes associated with the immunoprecipitated promoters may be correlated with biological processes or known protein functions. The GO annotations for biological processes were found for 1933 of the genes related to the immunoprecipitated promoters (*p* = 1.1E-08), and annotations for molecular functions were found for 2022 (*p* = 6.4E-11). The biological processes represented within this dataset of putative HBc targets included: metabolic process (1404;*p* = 3.0E-08); primary metabolic process (1286; *p* = 1.1E-08); cellular metabolic process (1248; *p* = 4.1E-08); and biological regulation (1206; *p* = 1.7E-03) (Figure 1). In addition, a remarkable amount of genes were found to be associated with dsRNA fragmentation, and intermediate filament cytoskeleton organization (Figure 2); the fold-enrichment value for these two particular groups of genes was 3.495 (*p* = 1.5E − 03) and 3.0749 (*p* = 1.0E − 03), respectively. The ten molecular functions of the encoded gene products that were most represented within the dataset of putative HBc targets are listed in Table 2. Protein binding was associated with the largest amount of putative HBc targets (1398, *p* = 9.7E-13), followed by catalytic activity (881, *p* = 2.9E − 06) and transcription regulator activity (289, *p* = 2.4E − 06). Further analysis suggested that one of the highly enriched gene promoters was related to an encoded protein product with MAP kinase phosphatase activity and another one was related to a protein with acylglycerol O-acyltransferase activity; the fold-enrichment value of these two was, respectively, 3.5303 (*p* = 1.4E − 03) and 3.4967 (*p* = 6.8E − 04) (Figure 3).

**Table 2:** The top ten molecular functions associated with HBc-immunoprecipitated host promoter sequences

**Molecular function**	Count*	*p*-value**	FDR***
Binding	2022	6.4000E-11	2.5632E-08
Protein binding	1398	9.7000E-13	7.7697E-10
Catalytic activity	881	2.8000E-06	2.8035E-04
Ion binding	702	9.4800E-03	1.3559E-01
Cation binding	697	4.9800E-03	8.3437E-02
Metal ion binding	692	4.2500E-03	8.3030E-02
Nucleotide binding	392	4.9000E-04	2.0657E-02
Purine nucleotide binding	339	6.2000E-04	02.4831E-02
Transferase activity	337	2.0000E-08	5.3400E-06
Ribonucleotide binding	328	3.6000E-04	1.7434 E-02
Transcription regulator activity	289	2.4000E-06	2.7463E-04

* Number of genes associated with the corresponding molecular function

** Significance testing value of genes associated with the corresponding molecular function.

*** False discovery rate of genes associated with the corresponding molecular function, as determined by the Benjamini & Hochberg (1995) testing method.

**Figure 4:**
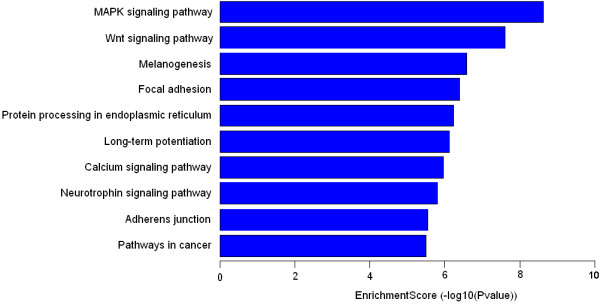
**The significant pathway bar plot of designated gene.** The bar plot shows the top ten Enrichment score (−log10(Pvalue)) value of the significant enrichment pathway

Pathway analysis was performed for all genes related to the promoters that immunoprecipitated with the HBV core protein. The 3100 genes were found to represent 55 pathways of the KEGG, according to the molecular interactions, reactions, and relations of the genes (see Additional file 5). Table 3 lists the top ten pathways corresponding to the significantly enriched promoters. The Enrichment score for pathways involved in tumor pathogenesis, MAPK signaling and Wnt signaling, were 8.5732 (*p* = 2.7E-09) and 7.5542 (*p* = 2.8E-08), respectively (Figure 4).

**Table 3:** The top ten pathways associated with HBc-immunoprecipitated host promoter sequences with the highest enrichment scores

**Pathway**	Selection counts*	*p*-value**	FDR***	Enrichment score****
MAPK signaling	64	2.6720E-09	5.8516E-07	8.5732
Wnt signaling	41	2.7910E-08	3.0561E-06	7.5542
Melanogenesis	30	2.9928E-07	2.1847E-05	6.5239
Focal adhesion (human)	47	4.6332E-07	2.5367E-05	6.3341
Protein processing in endoplasmic reticulum	41	6.5163E-07	2.8541E-05	6.1860
Long-term potentiation	23	8.2838E-07	3.0236E-05	6.0818
Calcium signaling	42	1.2489E-06	3.9074E-05	5.9034
Neurotrophin signaling	33	1.7810E-06	4.8755E-05	5.7494
Adherens junction	23	3.1867E-06	7.7544E-05	5.4967
Pathways in cancer	64	3.5835E-06	7.8478E-05	5.4457

* Number of genes associated with the corresponding pathway.

** Enrichment *p*-value of the corresponding pathway as determined by Fisher’s exact test.

*** False discovery rate of the corresponding pathway.

**** Enrichment score value of the corresponding pathway, expressed as -log10(*p*-value).

**Figure 5:**
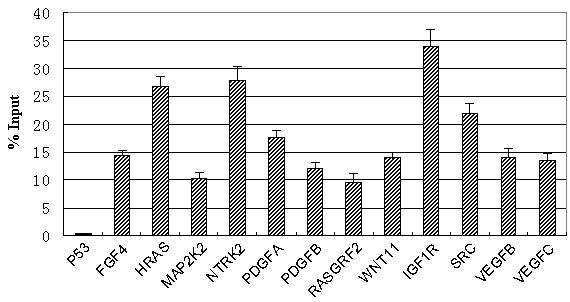
**The results of ChIP-qPCR analysis of HBV core protein binding to host gene promoters.** The promoter sequence percent of output DNA to input DNA (% input) was detected using qPCR of ChIP samples. The p53 gene promoter was used as the negative control. Data are expressed as mean ± SD of individual samples from three independent experiments

In order to verify the reliability of the ChIP-on-chip results, qPCR was used to detect the promoter sequences of selected genes in ouput DNA fractions from CHB patients and healthy controls. Twelve genes were selected for this analysis based upon known functions in tumor pathogenesis, and included: fibroblast growth factor 4 (FGF4), v-Ha-ras Harvey rat sarcoma viral oncogene (HRAS), mitogen-activated protein kinase kinase 2/MEK2 (MAP2K2), neurotrophic tyrosine kinase receptor 2 (NTRK2), platelet-derived growth factor A chain (PDGFA), platelet-derived growth factor B chain (PDGFB), Ras guanine nucleotide-releasing factor (RASGRF2), wingless-type MMTV integration site family (WNT11, a member of the Wnt superfamily of secreted glycoproteins), type 1 insulin-like growth factor receptor (IGF1R), proto-oncogene tyrosine-protein kinase (SRC), vascular endothelial growth factor-B (VEGFB), and vascular endothelial growth factor-C (VEGFC). The results indicated that the promoters of these 12 genes *(p* < 0.05) were immunoprecipitated by anti-HBc, suggesting that HBc physically interacted with each. Moreover, we tested the promoter sequences that were negative in HBc-ChIP-chip screening, such as the promoter of the tumor protein 53 (p53) gene. As shown in Figure 5, the highest percent of output to input (% Input) occurred with IGF1R (34.3%), followed by NTRK2 (27.9%), HRAS (26.8%), SRC (22.0%), VEGFC (19.0%), PDGFA (17.7%), FGF4 (14.4%), VEGFB (14.2%), WNT11 (14.0%), PDGFB (12.1%), MAP2K2 (10.2%), and RASGRF2 (9.4%). In addition, qPCR analysis further confirmed that HBc did not bind to the p53 promoter, as the p53% input was negative.

### HBV core protein binding to host gene promoters disrupts normal cellular gene expression

**Figure 6:**
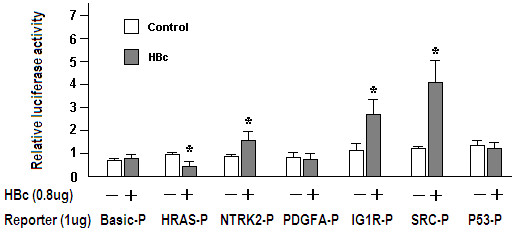
**Effects of HBV core protein binding to host gene promoters.** Relative luciferase activity was measured in cells co-transfected with one of six tumor pathogenesis-associated genes and the HBc gene. The pGL3-basic plasmid was used as the negative control. The vertical axis represents the relative proportion of luciferase activity of (cells co-transfected with HBc expression vector)/(cells transfected without HBc expression vector). Data are expressed as mean ± SD, *p < 0.05. Basic-P, pGL3-basic vectors; HRAS-P, NTRK2-P, PDGFA-P, IGF1R-P, SRC-P and P53-P represent the pGL3-basic-constructed plasmids for the promoters of HRAS, NTRK2, PDGFA, IGF1R, SRC and P53 genes, respectively

The above results suggested that HBV core protein can bind to host gene promoters, especially those involved in tumor pathogenesis. To investigate whether HBc binding regulated the transcriptional activity of these host cellular genes, reporter-gene expression assays were carried out. The promoter sequences of five genes (HRAS, NTRK2, PDGFA, IGF1R, and SRC) with >15% of output to input, and the promoter sequences of p53 as a negative control were selected for cloning into expression vectors and subsequent analysis. The sequences of the above promoters are listed in Additional file 3. As shown in Figure 6, the expression of all genes, with the exception of PDGFA and p53, was significantly changed in the presence of HBc, as evidenced by differential luciferase activity in HBc co-transfected cells and cells without the HBc expression vector. For SRC, IGF1R, and NTRK2, HBc co-transfection led to significant greater gene expression (luciferase activity fold-change: SRC, 3.5; IGF1R, 2.7, NTRK2, 2.1; *p* < 0.05). In contrast, HBc co-transfection led to significantly less expression of HRAS (luciferase activity fold-change: 0.4; *p* < 0.05). The HBc co-transfection slightly decreased the transcriptional activity of the PDGFA and p53 genes, whereas no statistical differences were detected (luciferase activity fold-change: PDGFA, 0.9, p > 0.05; P53, 0.9, p > 0.05).

## Discussion

In our study, we used the GenomePlex® Whole Genome Amplification ChIP-chip analyses and found that HBc can bind to more than three-thousand promoters in the human genome. Among this set of gene promoters, the high CpG density promoters were the most common. By evaluating the corresponding the GO annotations and the KEGG pathways for genes affiliated with each of these promoters, we determined that HBc tended to target the regulatory regions of genes with molecular function and malignant transformation in the liver cell repertoire. These results were consistent with our previous data showing that HBc preferentially binds CpG islands within the HBV covalently closed circular DNA [25]. To our knowledge, our current study is the first to provide evidence that HBc can bind to a large number of host gene promoters throughout the whole human genome. Our findings may provide new insights towards understanding the mechanisms underlying the ability of HBc to regulate host gene transcription.

The ChIP technique has proven to be a powerful tool for detecting protein-DNA interactions in living cells, and it is currently the gold standard procedure for identifying target genes of a given transcription factor of interest. Over the last several years, great strides have been made in expanding the use of ChIP from a one gene-at-a-time approach to a global type of analysis by hybridizing samples to genomic microarrays (i.e., ChIP-on-chip assay) [26]. In such a study, Dere *et al.* previously identified 1896 of the 2,3,7,8-tetrachlorodibenzo-p-dioxin (TCDD)-responsive genes in mouse liver by using microarray analysis [27]. Most regions were enriched 5.7-fold with the fold-enrichment values ranging from 1.7- to 111.4-fold [27]. Using ChIP-chip technique, Koudritsky and Domany showed that the number of target genes of each studied transcription factor (TF) ranged from a few hundred to several thousand [28]. Consistent with these data, 3100 HBc-immunoprecipitated host promoter regions were found to be enriched by at least 2-fold in HBc-infected tissues (*p* < 0.05, compared with control). Moreover, 2883 of 3100 gene promoters were found in all of the four pooled samples investigated by ChIP-on-chip, which indicates that this approach has a good level of repeatability and the reliability. In our study, arrays representing 18028 promoter regions and CpG islands of the whole human genome were used in combination with HBV HBc-based ChIP to identify putative targets of this viral factor. We also identified 266 HBc-bound gene promoters that were different among the four pooled samples. Such apparently inconsistent findings may reflect background differences among the pooled ChIP samples. We found it necessary to use pooled samples for the ChIP-on-chip assays since none of our ChIP samples provided enough DNA for genomic microarray analysis. The practice of pooling ChIP samples for ChIP-on-chip is very common, but is known to produce very high background when the samples are analyzed on genomic tiling arrays [29]. To verify the reliability of our ChIP-on-chip results, the method of ChIP-qPCR was used to detect the promoter sequences of randomly selected genes in samples of our output DNA. Our results confirmed that HBc can bind to the promoters of all 12 genes evaluated, including the MxA gene, which has been demonstrated as a *bona fide* target of HBc and was used as a positive control in our study [12,14].

The underlying mechanisms of the interaction between HBc and the human genome are poorly understood. HBc is a 183 residue protein with two domains: the N-terminal 144 amino acid domain that is sufficient for self-assembly into capsid particles, and the C-terminal arginine-rich domain, which shares a high similarity to protamine and functions as a nucleic acid-binding domain [16,30]. Although the C-terminal arginine-rich domain has been identified as imporatnt for HBc binding to pregenome RNA or genome DNA [31], no specific target sequence for the DNA-binding domain of HBc has been determined. Thus, while we know that the C-terminal arginine-rich domain can bind HBV-specific nucleotides *in vivo*, we have yet to determine the mechanisms mediating this binding specificity. However, based on the fact that HBV core particles specifically bind to the HBV pregenome or genome *in vivo*, we inferred that the binding specificity to the human genome might be mediated by the C-terminal arginine-rich domain, along with other regulatory regions in the N-terminal assembly domain. Previous studies have shown that a signal for nuclear transport is located near the carboxy termini of HBc, in the arginine-rich domain. This signal is comprised of a set of two direct PRRRRSQS repeats and is highly conserved among mammalian hepadnaviruses [32]. Recently, one study used a novel GST-fusion protein-based gel shift method to demonstrate that the C-terminal arginine-rich domain alone is capable of binding to DNA in a sequence-independent manner [33]. In addition, another study showed that HBc can enhance host NF-kB DNA-binding ability [34], which suggested that HBc may bind to and interact with other host nuclear proteins to enhance or inhibit their transcriptional activator functions. The large number of potential binding targets of HBc that were identified in our genome-wide analysis of human promoters supports the notion that HBc may utilize more than one of these proposed mechanisms. However, further study is needed to clarify this issue.

Previous studies have shown that HBV DNA integration and/or expression of HBV proteins may have direct effects on host cellular functions [35]. However, the roles of interaction between HBV proteins, especially HBc, and the human genome have not been well studied. Locarnini *et al*. [36] examined the effect of the HBV core protein on cellular gene expression in the hepatoma Huh-7 cell line by using a commercial high-density oligonucleotide array (Affymetrix Hu95A GeneChip). They found that only five genes had differential mRNA expression that was greater than three-fold at day 7 post-HBc expression. Among these five genes, four were down-regulated (by 3 ~ 15-fold) and included genes whose encoded products affect intermediary metabolism, cell surface receptors, and intracellular signaling. The fifth, a cytokine gene, was up-regulated. In contrast, our study utilized a microarray containing the genome-wide set of human gene promoters to search for potential binding sites of HBc, and identified over 3000 putative functional targets. Even though the full panel of these promoters has yet to be confirmed, the diverse spectrum of genes represented suggests that HBc may have multiple functions in regulating HBV pathogenesis and survival in the human host.

It is likely that HBc binding does not exert significant regulatory effects on every gene for which its promoter is bound. For some or many genes, HBc may merely mediate a slight modulation of the transcriptional activity. However, the cumulative effect of slight transcriptional modifications across the genome may impact overall cellular function. In our study, we found that HBc can bind to 64 gene promoters of the MAPK pathways, and 41 gene promoters of the Wnt/β-catenin signaling pathways. These two pathways are known to be critically involved in the development of HBV-related hepatocellular carcinoma. The MAPK pathways regulate a variety of normal human cellular activities, including proliferation, differentiation, survival, and death. As such, the components of MAPK signaling play a key role in several steps of tumorigenesis, including cancer cell proliferation, migration, and invasion [37]. Meanwhile, the Wnt/β-catenin signaling pathway has emerged as a critical player in both the development of normal liver and as an oncogenic driver in hepatocellular carcinoma [38]. As described above, the accumulation of slight effects from HBc binding to many gene promoters may produce sizeable effects on host cellular functions, possibly increasing a cell’s susceptibility to harmful factors, such as carcinogens [39]. Certainly, further studies will be needed to verify this theory and define the detailed factor(s) and step(s) that HBc works through, or that work through HBc, to regulate host gene transcription.

We investigated the functional relevance of HBV core protein binding to five of the genes whose promoters were identified in the ChIP-on-chip analysis. Transcriptional activity assays indicated that SRC, IGF1R, and NTRK2 genes were up-regulated by more than 2-fold when co-transfected with HBc. The HRAS gene, however, was down-regulated in the presence of HBc. SRC is the best characterized member of the family of nine tyrosine kinases that regulates cellular responses to extracellular stimuli, and activated mutants of SRC are oncogenic [40]. Among these four genes, SRC showed the greatest change in gene expression in HBc co-transfections, which may be related to the SRC gene promoter sequence that contains a potential NF-κB binding sequence (5′-GGGAAAATCC-3′). Thus, HBc might enhance the NF-κB DNA-binding ability on SRC [34]. However, it should be noted that the possible roles of the HBV core protein to the transcriptional activity of only small number of gene promoters were evaluated in our study; more sensitive and accurate high-throughput DNA-protein interaction and gene expression analysis techniques should be used in further studies.

## Conclusion

In conclusion, regardless of the mechanisms, the data of our study clearly demonstrate that HBc not only has the ability to bind to a large number of gene promoters in the human genome, but also has the ability to disrupt cellular gene expression of HBV-infected hepatocytes. Although further studies are needed to investigate the possible role of HBc in regulating the transcriptional activity of all cellular genes that HBc can bind, our study provides key insights into the detailed factor(s) and step(s) that HBc works through, or that work through HBc, to regulate host gene transcription. This information provides a new perspective towards understanding the mechanisms of HBV-related liver diseases, such as liver chronicity, fibrogenesis, and carcinogenesis. Manipulation of the transcriptional profile in HBV-infected hepatocytes may represent a key pathogenic mechanism of HBV infection.

## Competing interests

The authors declare that they have no competing interests.

## Authors’ contributions

YG designed this study, performed the ChIP experiment, reporter gene analysis, and statistical analysis, and prepared this manuscript. WK and YL collected clinical samples and patient information. WK examined the HBV DNA level and HBV indexes from serum samples and also revised the manuscript. XL and AX performed the qPCR experiment and analyzed the data. YL, JZ, and JZ participated in the reporter gene analysis. ZY helped with manuscript revision. All authors read and approved the final manuscript.

## Financial disclosure

This study was supported by grants from the 11th Five-Year Plan of China: the Project for Prevention and Treatment of AIDS, Viral Hepatitis and Other Major Infectious Diseases (No. 2009ZX10004-311-004), the Natural Science Basic Research Program of Shaanxi Province of China (No. 2009JM4005) and the National Natural Science Foundation of China (No. 31271394).

## Supplementary Material

Additional file 1 Primers of HBV DNA for PCR amplification and PCR products. Click here for file

Additional file 2 Primers of qPCR. Click here for file

Additional file 3 The location and sequence list of promoters which were detected by qPCR to verify the reliability of the ChIP-on-chip results. Click here for file

Additional file 4 The Results of ChIP-on-chip analysis. Click here for file

Additional file 5 The Result of pathway analysis. Click here for file
