# Supplementary material for: Hepatitis B viral core protein disrupts human host gene expression by binding to promoter regions
Source: BMC Genomics. 2012 Oct 22;13:563. doi: 10.1186/1471-2164-13-563 (PMC3484065; doi:10.1186/1471-2164-13-563)
Supplement: Additional file 1 — Primers of HBV DNA for PCR amplification and PCR products. [file 1471-2164-13-563-S1.doc]

Additional file 1. Primers of HBV DNA for PCR amplification and PCR products

| Primer No. | Primer Sequence and Location, 5’-3’* | Product Size, bp |
| --- | --- | --- |
| 1 | forward: tccacaacattccaccaagc 2 nt - 21 nt | 620 |
|  | reverse: caggatgatgggatgggaat 621 nt - 602 nt |  |
| 2 | forward: cctgctcaaggaacctctatg 533 nt - 553 nt | 652 |
|  | reverse: aacacttggcagagacctga 1184 nt - 1165 nt |  |
| 3 | forward: ggctttcactttctcgccaa 1086 nt - 1105 nt | 652 |
|  | reverse: cctcccagtccttaaacaaac 1737 nt - 1717 nt |  |
| 4 | forward: tcttgcccaaggtcttacataag 1636 nt - 1658 nt | 654 |
|  | reverse: agaggagtgcgaatccaca 2289 nt - 2271 nt |  |
| 5 | forward: cacatttcctgtcttacctttgg 2210 nt - 2232 nt | 651 |
|  | reverse: accaacctcccatgctga 2860 nt -2843 nt |  |
| 6 | forward: agagagagactacacgcagt 2782 nt - 2801 nt | 534 |
|  | reverse: agtcggaacagggtttactg 100 nt - 81 nt |  |

*The numbers shown represent each primer’s location start site from the unique *EcoR*I site in HBV DNA. PCR products overlap by 79 bp to 99 bp.


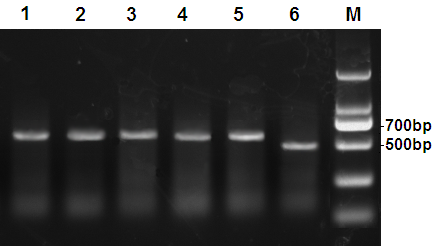


Six PCR products of HBV DNA.

Lane 1-6: PCR products of HBV DNA were amplified using primer pairs No.1-6 in above table, respectively. M: DL2000 DNA maker. PCR products (10ul/well) were electrophoresed on a 1.5% agarose gel and visualized with ethidium bromide staining.
