# Supplementary material for: Hepatitis B viral core protein disrupts human host gene expression by binding to promoter regions
Source: BMC Genomics. 2012 Oct 22;13:563. doi: 10.1186/1471-2164-13-563 (PMC3484065; doi:10.1186/1471-2164-13-563)
Supplement: Additional file 2 — Primers of qPCR. [file 1471-2164-13-563-S2.doc]

Additional file 2. Primers of qPCR

| promoter | Primer sequence(5’-3’) | Region of PCR | Product Size |
| --- | --- | --- | --- |
| 1.FGF4 promoter | LEFT PRIMER:CTGTGTCAGCTCCAGGGAAG  RIGHT PRIMER:AGCCTCTGTGTCCTCTCACC | chr11:69298867-69299061 | 195bp |
| 2. HRAS promoter | LEFT PRIMER: GCCTGGAGACAAAGCAAGAC  RIGHT PRIMER: GCCTGGCCTGATTTTTGTAT | chr11:525067-525265 | 199bp |
| 3.MAP2K2 promoter | LEFT PRIMER: CTGGGCAACATAGTGAGACC  RIGHT PRIMER: CAGAGCAAGACTCTGTCTCCAA | Chr19:4074807-4075011 | 205bp |
| 4. NTRK2 promoter | LEFT PRIMER: CCAGGCTAGAGTGCAATGGT  RIGHT PRIMER:CCTGAGGTCAGGAGTTCGAG | chr9:86474488- 86474684 | 197 bp |
| 5. PDGFA promoter | LEFT PRIMER: GAGTGAGTGTGCCTGCATGT  RIGHT PRIMER: CTTTTCCTGTGGTGTCTTTGG | chr7:526244-526448 | 205 bp |
| 6. PDGFB promoter | LEFT PRIMER: GAGACCACCAAGCTGGAAAG  RIGHT PRIMER:GCAGCGTGATTTCATCACTT | chr22:37968068-37968269 | 202 bp |
| 7.RASGRF2 promoter | LEFT PRIMER:CTCAGGCTCCCAAGTAGCTG  RIGHT PRIMER:TCCTGACTTTGCTTTACTAAGCCTAT | chr5:80292533-80292736 | 204 bp |
| 8. WNT11 promoter | LEFT PRIMER:ACTGCCAGTTTTCCAAAGTGA  RIGHT PRIMER:ATAGACTTTTTGCTTACAAGGCTCA | chr11:75595052-75595246 | 195 bp |
| 9. IGF1R promoter | LEFT PRIMER:TGTTCTAAAGATTCGGTCACAGC  RIGHT PRIMER:CTCATTGACCTGCCTTGCTT | chr15:97008106-97008304 | 199 bp |
| 10. SRC promoter | LEFT PRIMER: TGTGTGTGTGAGAAAACACAAAAT  RIGHT PRIMER:TCAGTCCTCCCAGGATGTTC | chr20: 35408027-35408230 | 204 bp |
| 11. VEGFB promoter | LEFT PRIMER:ATGTTACGGGCGGTTGTAAG  RIGHT PRIMER:GATGACTCCGTGGGAAGAAC | chr11:63758718-63758914 | 197 bp |
| 12. VEGFC promoter | LEFT PRIMER:GAACGATGTACCATTTCATTATTCA  RIGHT PRIMER:CAGCAAAGGATGGTGTAGCA | chr4:177950535-177950731 | 197 bp |
| 13. P53 promoter 1 | LEFT PRIMER:GAGTGCAGTGGCACGATTT  RIGHT PRIMER:GAATCGCTTTCAGCTCAGGA | chr17:7564817-7565011 | 195bp |
