# Supplementary material for: Hepatitis B viral core protein disrupts human host gene expression by binding to promoter regions
Source: BMC Genomics. 2012 Oct 22;13:563. doi: 10.1186/1471-2164-13-563 (PMC3484065; doi:10.1186/1471-2164-13-563)
Supplement: Additional file 3 — The location and sequence list of promoters which were detected by qPCR to verify the reliability of the ChIP-on-chip results. [file 1471-2164-13-563-S3.doc]

**Additional file 3. The location and sequence list of promoters which were detected by qPCR to verify the reliability of the ChIP-on-chip results**

**1. FGF4 promoter region sequence**

Location chr11:69298852-69299101, 249bp

TACCTGCTGTGAGCCCTGTGTCAGCTCCAGGGAAGGGAGGGTGCTCTTGGGGTGCCCCCTCACTCCTTAGAACCTGCGTTCTGCAGCTTCGAGATAGAGAGCGACCTCAGAACACTCAGCCAAGAAACAGACAGGCCAGGGCATGGCCTCCAGACAGGAGGTCATGGGAGAAGGAGAGTCAGCGCTGCCCGGTGAGAGGACACAGAGGCTCTGTGGGAGAAGGGAGGGACAGTCCCGGCATGAGTACACA

**2. HRAS promoter region sequence**

Location：chromosome 11:525050-525299，249bp

CTCACCGCTGCACTCCAGCCTGGAGACAAAGCAAGACTCCGTCTCAAAAAAAAAAAAAAATAGACAACTTGGGGCCGGGCGCGGTGGGTCACACCTGTAATCCCAGCACTTTGGGAGGCCGAGGCGGGCAGATCACCTGAGGTCGGGAGTTTGAGACCAGCCTGGCCAACATGGTGAAACCCTGTCTCTACTAAAAATACAAAAATCAGGCCAGGCACGGTGGCTCACGCCTGTAATCCCAGCACTTTGG

**3. MAP2K2 promoter region sequence**

Location：chromosome 19: 4074726-4075075， 349bp

ACGGTGGCTCACGTCTGTAATCCCAGCATCTTGGGAGGCCAAAGCTAAAGGATCGCTTGAGCCCAGGAGGTCAAGATCAGCCTGGGCAACATAGTGAGACCCTGTCTCTACCAAAAAAAAAAAAAAAGATCATGAAGATGATTTCTTCAAAGGAAGTGATGTTTGAGCAGAGATCTTTTCTCTCCTTTCTTTCTTTCTTTCTTTCTTTCTTTCTTTCTTTCTTTCTTTCTTTCTTTCTTTCTTTCTTTCTTTCTTTCTTTTATTTTGGAGACAGAGTCTTGCTCTGTCACCAGGCTGGAGGGCAGTGGCACGATCTCAGCTCACTCCAACCTCCGCCTCCTGGGTTCAAG

**4. NTRK2 promoter region sequence**

Location：chromosome 9: 86474485-86474834， 349bp

TCCCCAGGCTAGAGTGCAATGGTGCAACCTCAGCTCACTGCAACCTCCGCTTCCCAGGTTCAAGCGATTCTCCTGCTTCAGCCTCCCTGGTTGCTGGGATTACAGGTGCTCATCACCATGTGCAGCTAATTTTTTGTATTTTTAGTAGAGACAGGGTTTTACCATGTTGCCCAGGCTGGTCTCGAACTCCTGACCTCAGGTGATCCACCCACCTCAGCCCCCCAAAGTTCTGAGATTACAGACGTGAGACACTGCACCCGACCGGGGTGTTCATTTTCTAAAATCTTAAGTTGAATGCCTTTTAAAAAAAAAAAGTCTATTTAAACTCTGAGTTTACTGCCAAGAATTGC

**5. PDGFA promoter region sequence**

Location：chromosome 7:526207-526557， 350bp

TGTGTGAATGTGTGTCCCTGTGTGTGGACCTGCACGTGAGTGAGTGTGCCTGCATGTGAATGCGTGTGAATGCATGAGCCTGTGTGTGAATGTGTGAACCTGTATGTGAATGTGTGTGCCTGTACGTGAATGTGTGTGGTGCTCCATGTGTTTTTGTGTCTATGTGTTTGTCTCTGTGTGGCTGTAACTTTCTGTGACTCTCCAAAAACCCTGATTCAAAACCAAAGACACCACAGGAAAAGAGGACTACAGAACAATTTCCCACTTAAGCATGGACACAAAAATCCTTAACAAATGATTAACAAGTAGAATCAATATAAAAAGAAACGTATTTAATGACCAAGGTGGGTT

**6. PDGFB promoter region sequence**

Location：chromosome 22: 37967991-37968340，349bp

CACTTGCCCAGCAGCTTCATCCACCTCATGCCAACCCAGCTGCAGGTCTCAATCCCCCTGCCCACGTGAGGACGCAGGAGACCACCAAGCTGGAAAGTGGCAGAGCCAGAGCGCTCTGATCAAGGCCTCCCAGAGCTTCCCCACTCCCTGCCCTGTTTTGCTCTTATCCACCCCAACCCGAAGAGGCACAGGACGGAGGCTCAGTTTGCCCATCTATGAAATGGGCAGAGTGCTTCCTGCCCAGTTCCGCAAAGTGTTCAAGTGATGAAATCACGCTGCCCTCAGCAGAACCCCAGAAACAAAAATATGATATGGAAAATGACTGTATCAGAGGCACAAGATCTTGGCCC

**8. WNT11 promoter region sequence**

Location：chromosome 11: 75594922-75595271， 349bp

AGTAACCACCACCATAATAAATAAAGCTACTATGAATATTTAAATATAGGTCTTTGTGTTTCTTTTGGGTAAATCTCCAGGAATGGAATTGATGGGTCTAATGGTAAATGTATGTTGAAATTTATAAGAAACTGCCAGTTTTCCAAAGTGACTATAACATCCTGCATTCCCATCAGCAATGTATAATAGTTCTGTTTGTTTCACATCTTTGCTATCACTTGTGATTGCCAGTCTTTTTAAATTTTGAGGTGGTATCTCATTGTAGTTTTAATTTGAATTTTCCTAATGACAAAATTAAATTGAGCCTTGTAAGCAAAAAGTCTATTTGAGATCAGTCTCTCTCTCTCTCT

**9. IGF1R promoter region sequence**

Location：chromosome 15: 97008087-97008336，250bp

CTGGAATCATCAGAAACAATGTTCTAAAGATTCGGTCACAGCCACCACATTCGTAGAAATCCTGACTCACTAGATGGGATAAGAAAAGGAATAGGTATTTTTTTTTGAGAAAGTTCTGCAGAAGATGGTTCTGATGTGTAGTTGGGTTTAAACAATTTTAGAGCTTGCCTCTTGTCCTCAAAAGCTTAAACACAATTCAAGCAAGGCAGGTCAATGAGGAAAATAAGTACAGTAGTTATGTTTGCTTAGC

**10. SRC promoter region sequence**

Location：chromosome 20: 35408001-35408251， 250bp

ATGCATTGGAGGAAGAGGCTTTTTTTTGTGTGTGTGAGAAAACACAAAATAAATAAACACAGAACCAGGACACCACCACATTTGAGAGCAAATATCAGAGTTGGGAAACCACAGACGCGGACAGGAAGGCTTCCCTGGTAGGAAGCAGGGGGACAAGAAGCCAGTTACTGAGGTTACGGGAAAATCCGCACGAGGCAGCAGTGCTGGGAAGAACATCCTGGGAGGACTGAGGGCTGAATACTATAGTGAGC

**11. VEGFB promoter region sequence**

Location：chromosome 11: 63758641-63758990， 350bp

TGAAGCTCTTGGATCCCCTGTTCTCTCTGGGCGGGGACATTGTCTGCCTCACAGGGCAATTGTGAGGGTTGAAGGAGATGTTACGGGCGGTTGTAAGCAGCGGGTTACAAAGCTGCTCCTCTCCCCATACAGGGGGTGAGCTTCATTCATTCATTCCTCTTATGTCAGTGTCCTCCAGTGGGACCCCCCATGCCAAGGCCTGCCGCTCATGCTAGACCACCCTGGTAAAGTGGACAGGACAGGCTGGGCCCCAGGTTCTTCCCACGGAGTCATCAAACATTTGGATGTCCAGAGCATGCTGTCTAAAAGGCCTCTCTGTGCCCCCAGCACCCGCATTCTGTATGCTGTGC

**12. VEGFC promoter region sequence**

Location：chromosome 4: 177950389-177950738，349bp

AGTGTTCATTGAATGAGTTAGAAAATAGCTTAGAGATGATTTAGTCCCACCTCTTTCCCAAAGTTTAAATATCATTTACTATATCCCTGGGAGATGTTTATTAGTCACCACACCAACACTACCAGGGTCAGATAGCCTGATACTTAGAACGATGTACCATTTCATTATTCAACCTAACATTTCAAATATTTTTTTATGTTAGGCTTAAATTTTGCCTCTGAATCTTCTATACCCTACTCCCATTAGTCCTATTTTTACCCTCTGAAAAAAACTTATAATAAAACAATTTTTTATATGGCAACATTTCAATATTTGAAGATATCTGCTACACCATCCTTTGCTGTCTGTTC

**13. P53 promoter 1 region sequence**

Location：chromosome 17: 7564797-7565097，300bp

CCGCTGTGTCGTCCAGGCTGGAGTGCAGTGGCACGATTTCAGCTTACTGCAACCTCTGCCTCCCGGTTTCAAGGGATTCTCCTGCCTCAGCCTCTCGAGTAGCTGGGATTATAGGTATGCACTACCACGCCTGGCTAAGTTTTGTATTTTTAGTATAGACGGGGTTTTGCCATGTTGCCCAGACTGGTCCGGAACTCCTGAGCTGAAAGCGATTCACCCGCCTTGGCCTCCCAAAGTGCTGGGATTACAGGCGGGAGCCACCGTGCCCGGCCTCCAGTATTTTGTTTATTTATTTTTTTT
